# Evidence for a Clathrin-independent mode of endocytosis at a continuously active sensory synapse

**DOI:** 10.3389/fncel.2014.00060

**Published:** 2014-02-25

**Authors:** Michaela Fuchs, Johann Helmut Brandstätter, Hanna Regus-Leidig

**Affiliations:** Animal Physiology, Department of Biology, Friedrich-Alexander-University Erlangen-NurembergErlangen, Germany

**Keywords:** ribbon synapse, endocytosis, active zone, dynamin, endophilin, synaptojanin, piccolino, bassoon

## Abstract

Synaptic vesicle exocytosis at chemical synapses is followed by compensatory endocytosis. Multiple pathways including Clathrin-mediated retrieval of single vesicles, bulk retrieval of large cisternae, and kiss-and-run retrieval have been reported to contribute to vesicle recycling. Particularly at the continuously active ribbon synapses of retinal photoreceptor and bipolar cells, compensatory endocytosis plays an essential role to provide ongoing vesicle supply. Yet, little is known about the mechanisms that contribute to endocytosis at these highly complex synapses. To identify possible specializations in ribbon synaptic endocytosis during different states of activity, we exposed mice to controlled lighting conditions and compared the distribution of endocytotic proteins at rod and cone photoreceptor, and ON bipolar cell ribbon synapses with light and electron microscopy. In mouse ON bipolar cell terminals, Clathrin-mediated endocytosis seemed to be the dominant mode of endocytosis at all adaptation states analyzed. In contrast, in mouse photoreceptor terminals in addition to Clathrin-coated pits, clusters of membranously connected electron-dense vesicles appeared during prolonged darkness. These clusters labeled for Dynamin3, Endophilin1, and Synaptojanin1, but not for AP180, Clathrin LC, and hsc70. We hypothesize that rod and cone photoreceptors possess an additional Clathrin-independent mode of vesicle retrieval supporting the continuous synaptic vesicle supply during prolonged high activity.

## INTRODUCTION

At chemical synapses, exocytosis of transmitter-filled synaptic vesicles is followed by compensatory endocytosis to retrieve synaptic vesicle components from the plasma membrane and to restore the cell surface area. Multiple pathways contribute to compensatory endocytosis at chemical synapses, including Clathrin-mediated retrieval of single vesicles, bulk retrieval of large cisternae, and kiss-and-run retrieval (reviewed in Dittman and Ryan, 2009; Royle and Lagnado, 2010; Haucke et al., 2011; Saheki and de Camilli, 2012). In contrast to conventional chemical synapses, tonically active ribbon synapses support high and sustained rates of exocytosis (reviewed in Matthews and Fuchs, 2010; Mercer and Thoreson, 2011; Regus-Leidig and Brandstätter, 2012). Thus, compensatory endocytosis has to play an important role for vesicle replenishment at these synapses.

Among the different types of sensory neurons with ribbon synapses, photoreceptors display extraordinary high rates of sustained glutamate release, with highest release rates in the dark (Heidelberger et al., 2005). The ultrastructural hallmark of photoreceptor synapses, the presynaptic ribbon, is an electron-dense plate-like structure which extends several hundred nm into the cytoplasm and tethers a large number of transmitter vesicles via fine filaments (Rao-Mirotznik et al., 1995; Sterling and Matthews, 2005; tom Dieck and Brandstätter, 2006). The exact function of the synaptic ribbon in photoreceptor neurotransmission is not yet fully understood, but it is widely accepted that the synaptic ribbon ensures the precise spatial and temporal control of vesicle fusion, and accommodates the high rates of synaptic transmission (Schmitz, 2009; Regus-Leidig and Brandstätter, 2012).

Studies on the mechanisms of endocytosis at photoreceptor ribbon synapses are rare, but there is growing evidence for Clathrin-mediated endocytosis (CME) as a predominant mechanism. Early tracer uptake studies on cone photoreceptors from different species showed labeling of single vesicles, some of which were coated (reviewed in LoGiudice and Matthews, 2007), and a few electron microscopical studies on rat, guinea pig, and mouse photoreceptor terminals showed Clathrin-coated pits (CCP) or Clathrin-coated vesicles (CCV; Gray and Pease, 1971; Spiwoks-Becker et al., 2001; Bai et al., 2006; Zampighi et al., 2011). In addition, recent immunocytochemical studies on various species report the presence of Clathrin, Dynamin, Amphiphysin, Endophilin, and Synaptojanin in photoreceptor terminals (Sherry and Heidelberger, 2005; Xi et al., 2007; Holzhausen et al., 2009; Grossman et al., 2013; Wahl et al., 2013). All these studies were performed on light adapted retinae, when photoreceptors are minimally active. Since photoreceptors release the highest amounts of glutamate in the dark, it is conceivable that they developed specialized adaptations to increased vesicle turnover rates during darkness.

In this study, we exposed mice to a controlled light regime with light adaptation followed by different durations of dark exposure and compared the distribution of endocytotic proteins at rod photoreceptor, cone photoreceptor, and bipolar cell ribbon synapses in the differently adapted retinae with light and electron microscopy. Based on our findings, we propose that in addition to CME, photoreceptors possess a Clathrin-independent mode of compensatory vesicle retrieval during prolonged, maximal activity.

## MATERIALS AND METHODS

### ETHICS STATEMENT

All animal experiments were approved and registered by the county’s animal welfare authorities (permit number: 54-2531.31-15/06; Regierung von Mittelfranken, Ansbach, Germany) and performed in compliance with the guidelines for the welfare of experimental animals issued by the Federal Government of Germany and the FAU Erlangen-Nuremberg. Mouse breeding was performed in the animal facilities of the FAU Erlangen-Nuremberg according to European and German (Tierschutzgesetz) guidelines for the welfare of experimental animals (AZ 820-8791.2.63).

### ANIMALS AND LIGHT REGIME

Adult (age 4 months) male Dark Agouti rats (kindly provided by the Franz-Penzoldt-Zentrum, Erlangen), adult (2–3 months) male and female C57BL/6JRj (BL/6) mice, BsnΔEx4/5 mice (Altrock et al., 2003; kindly provided by E. D. Gundelfinger, Leibniz Institute for Neurobiology, Magdeburg, Germany), Tg(Rac3-EGFP)JZ58Gsat/Mmcd (Rac3-EGFP) mice, and Tg(Lrrc55-EGFP)KS290Gsat/Mmcd (Lrrc55-EGFP) mice were used in this study. The latter two strains were obtained from the Mutant Mouse Regional Resource Center, a NCRR-NIH funded strain repository, and were donated to the MMRRC by the NINDS funded GENSAT BAC transgenic project. Both strains were back-crossed to a BL/6 background.

All animals were kept in a 12/12 h light/dark cycle with light on at 6:00 a.m. and an average illumination of 200 lux (white light; TLD 58W/25 tubes, Philips, Hamburg, Germany). Light and dark adaptation was performed as follows: mice were light adapted for 3 h or light adapted for 3 h and subsequently exposed to different periods of darkness (1 min, 15 min, 3 h, 15 h).

### RETINA PREPARATION FOR LIGHT MICROSCOPY AND LIGHT MICROSCOPIC ANALYSIS

Preparation of retinal tissue and antibody incubation for light microscopic immunocytochemistry (ICC) was performed as described previously (tom Dieck et al., 2005; Regus-Leidig et al., 2009). Dark adapted retinae were prepared and fixed under dim red light. Briefly, the eyes were opened and retinae were immersion fixed in the eyecup for 15 or 30 min in 4% paraformaldehyde (PFA) in phosphate buffer (PB; 0.1 M, pH 7.4). Retinae were mounted in freezing medium (Reichert-Jung, Bensheim, Germany), and 12 μm thick vertical sections were cut with a cryostat (Leica CM3050 S, Leica, Wetzlar, Germany). Primary antibody incubation was carried out overnight at room temperature, secondary antibody incubation for 1 h. For analysis, labeled sections were examined with a Zeiss Axio Imager Z2 equipped with an ApoTome (Zeiss, Oberkochen, Germany). Images were taken with a 20× (0.8, Apochromat) or a 100× (1.3 oil, Plan-Neofluar) objective as stacks of multiple optical sections, and projections were calculated with the ZEN blue 2012 software (Zeiss). Images were adjusted for contrast and brightness using Adobe Photoshop CS (Adobe, San Jose, CA, USA). 3D reconstructions from z-stacks were generated with Imaris software (Bitplane, Zurich, Switzerland). Colocalization of immunofluorescent puncta was determined by using the custom-made analyzing software “OpenView” on projections of z-section stacks. Rectangular regions (4 × 4 pixel) centered on individual Dynamin-puncta were automatically drawn and compared with the fluorescent staining of the respective second channel (i.e., staining for Endophilin or Synaptojanin1; software written by N. E. Ziv, The Rappaport Family Institute for Research in Medical Sciences, Department of Physiology and Biophysics, and The Lorry Lokey Interdisciplinary Center for Life Sciences and Engineering, Technion-Israel Institute of Technology, Haifa, Israel; Regus-Leidig et al., 2010b). For each adaptation state and antibody combination, 3 images from 3 retinal sections per mouse (*n* = 3) were analyzed.

### RETINA PREPARATION FOR ELECTRON MICROSCOPY

For conventional electron microscopy, retinae were fixed in 4% PFA and 2.5% glutaraldehyde for 2 h at room temperature. Tissue contrasting was carried out by incubation in 1.5% potassium ferrocyanide and 2% osmium tetroxide in 0.1 M cacodylate buffer (pH 7.4) for 1.5 h. Retinae were dehydrated using an ethanol series and propylene oxide with 0.5% uranyl acetate added at the 70% ethanol step. The tissue was embedded in Renlam resin (Serva, Heidelberg, Germany).

For pre-embedding immunoelectron microscopy, retinae were prefixed in 4% PFA in Soerensen buffer (0.1 M Na_2_HPO_4_·2H_2_O, 0.1 M KH_2_PO_4_, pH 7.4) for 50 min at room temperature and further processed as described previously (Brandstätter et al., 1996). Briefly, after four cycles of freezing in liquid nitrogen and thawing at 37°C, retinae were PBS washed and embedded in buffered 2% Agar. Agar blocks were sectioned in 100 μm sections with a vibratome (Leica VT 1000 S, Leica, Wetzlar, Germany). The sections were incubated in 10% normal goat serum, 1% bovine serum albumin in PBS for 2 h, followed by incubation with primary antibodies for 4 days at 4°C. PBS washed sections were incubated with biotinylated secondary antibodies, and visualized by Vectastain ABC-Kit (both from Vector Laboratories, Burlingame, CA, USA). The sections were fixed in 2.5% glutaraldehyde in cacodylate buffer. Diaminobenzidine precipitates were silver enhanced and postfixed in (1.5% potassium ferrocyanide and) 0.5% OsO_4_ in cacodylate buffer for 30 min at 4°C. Dehydrated specimens were flat-mounted in Epon resin (Fluka, Buchs, Switzerland).

Ultrathin sections (58 nm) were stained with uranyl acetate and lead citrate. The sections were examined and photographed using a Zeiss EM10 electron microscope (Zeiss, Oberkochen, Germany) and a Gatan SC1000 Orius TM CCD camera (GATAN, Munich, Germany) in combination with the Digital Micrograph 3.1 software (GATAN, Pleasanton, CA, USA). Images were adjusted for contrast and brightness using Adobe Photoshop CS5 (Adobe, San Jose, CA, USA). 3D reconstructions from serial sections were created with Reconstruct v1.1.0.0 (J. Fiala, Medical College of Georgia). Vesicle diameters were measured using ImageJ (Schneider et al., 2012).

### ANTIBODIES

The following antibodies were used for ICC and pre-embedding immunoelectron microscopy (pre-EM): monoclonal mouse anti-Calbindin (ICC 1:2,000; #214011 Synaptic Systems, Göttingen, Germany), mouse anti-Clathrin light chain (Clathrin LC; ICC 1:1,000; #113011 Synaptic Systems), mouse anti-Dynamin (ICC 1:10,000; #ADI-VAM-SV041-E Enzo Life Sciences, Farmingdale, NY, USA), mouse anti-uncoating ATPase (hsc70; ICC 1:100; #149011 Synaptic Systems), polyclonal rabbit anti-Amphiphysin (ICC/pre-EM 1:10,000; #120002 Synaptic Systems), rabbit anti-AP180 (ICC 1:10,000; #155003 Synaptic Systems), rabbit anti-Caveolin1 (ICC 1:1000; #161003 Synaptic Systems), rabbit anti-Dynamin3 (ICC/pre-EM 1:10,000; #115302 Synaptic Systems), rabbit anti-Endophilin1 (ICC/pre-EM 1:10,000; #159002 Synaptic Systems), rabbit anti-Synaptojanin1 (ICC 1:5,000; pre-EM 1:10,000; #145003 Synaptic Systems), rabbit anti-RIBEYE A-domain (ICC 1:50,000; #192103 Synaptic Systems; Regus-Leidig et al., 2013), rabbit anti-Velis-3 (ICC 1:5,000, #51-5600 Invitrogen/Life technologies, Carlsbad, CA, USA; Regus-Leidig et al., 2010a), guinea pig anti-GFP (ICC 1:400; this antibody was generated and affinity-purified as described in Mühlhans et al., 2011. Peptide immunization of guinea pigs was performed by Pineda Antikörper-Service, Berlin, Germany), guinea pig anti-Piccolo 44a (ICC 1:4,000; Dick et al., 2001), guinea pig anti-VGluT1 (ICC 1:50,000, #AB5905 Millipore; Regus-Leidig et al., 2009). All Synaptic Systems antibodies were tested for specificity by the manufacturer, and western blot images and immunocytochemical stainings showing the specificity are available on the Synaptic Systems website.

The following secondary antibodies were used: Alexa^TM^ 488 goat anti-guinea pig, goat anti-mouse and goat anti-rabbit IgG conjugates (1:500; Molecular Probes, Eugene, OR, USA), Cy3 goat anti-mouse and goat anti-rabbit IgG conjugates (1:200; Dianova, Hamburg, Germany), Cy5 goat anti-guinea pig, goat anti-mouse and goat anti-rabbit IgG conjugates (1:100; Dianova).

## RESULTS

### DISTRIBUTION OF ENDOCYTOTIC PROTEINS IN THE MOUSE RETINA

**FIGURE 1:**
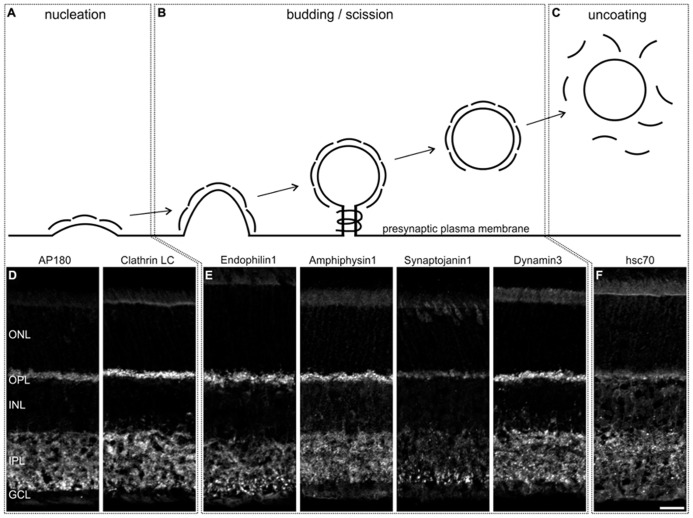
**Distribution of endocytotic proteins in a 3 h light adapted mouse retina.** **(A–C)** Schematic representation of the steps of Clathrin-mediated endocytosis. (**D–F)** Images of vertical cryostat sections through light adapted C57BL/6JRj retinae stained with antibodies against endocytotic proteins involved in these steps. ONL, outer nuclear layer; OPL, outer plexiform layer; INL, inner nuclear layer; IPL, inner plexiform layer; GCL, ganglion cell layer. Scale bar: 20 μm.

First, we stained vertical cryostat sections of light adapted C57BL/6JRj (BL/6) retinae for proteins involved in various modes of endocytosis, but particularly for proteins described in the different steps of CME (for a detailed review, see e.g., Saheki and de Camilli, 2012), i.e., nucleation (AP180, Clathrin LC; **Figures 1A,D**), budding and scission (Endophilin, Amphiphysin, Synaptojanin, Dynamin; **Figures 1B,E**), and uncoating (hsc70; **Figures 1C,F**). For all examined endocytotic proteins, we observed staining in both synaptic layers of the retina, the outer (OPL) and the inner (IPL) plexiform layer. Strong staining was detected for Clathrin LC, Endophilin1, Amphiphysin1, and Dynamin3 (**Figures 1D,E**), weaker staining for AP180 (**Figure 1D**), Synaptojanin1 (**Figure 1E**), and hsc70 (**Figure 1F**). Other modes than CME that are discussed for photoreceptor ribbon synapses include endocytosis via caveolae (Kachi et al., 2001; Kim et al., 2006). However, staining of retina sections with an antibody against Caveolin1 did not produce any specific staining in the two synaptic layers (data not shown).

**FIGURE 2:**
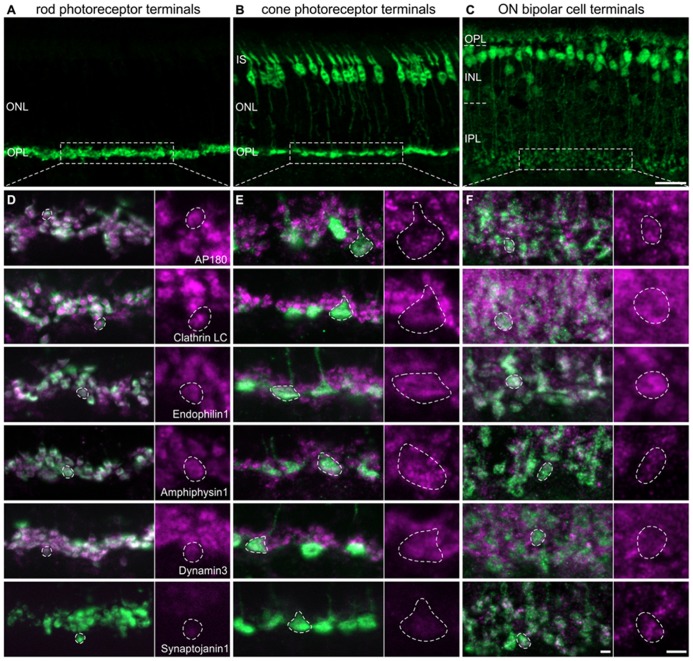
**Endocytotic proteins in terminals of 3 h light adapted mouse rod photoreceptors, cone photoreceptors, and ON bipolar cells. (A–C)** Images of vertical cryostat sections through C57BL/6JRj **(A)**, Rac3-EGFP **(B)**, and Lrrc55-EGFP **(C)** retinae stained with antibodies against VGluT1 **(A)** and GFP **(B,C)**. **(D)** Images of rod photoreceptor terminals of C57BL/6JRj retinae stained with antibodies against Velis-3 or VGluT1 (green) and endocytotic proteins (magenta). **(E)** Images of cone photoreceptor terminals of Rac3-EGFP retina stained with antibodies against GFP (green) and endocytotic proteins (magenta). **(F)** Images of ON bipolar cell terminals of Lrrc55-EGFP retina stained with antibodies against GFP (green) and endocytotic proteins (magenta). Single synaptic terminals are delineated with dashed lines. ONL, outer nuclear layer; OPL, outer plexiform layer; IS, inner segments; INL, inner nuclear layer; IPL, inner plexiform layer. Scale bar in **C** (for **A–C**): 20 μm, in **F** (for **D–F**): 2 μm.

For the detailed analysis of the presence and possible differences in the expression of CME components at different types of retinal ribbon synapses - rod photoreceptor, cone photoreceptor, and ON bipolar cell synapses – we double labeled vertical cryostat sections of light adapted retinae for the endocytotic proteins and respective cell markers. Rod photoreceptor terminals of BL/6 mouse retina were stained with antibodies against Velis-3 or VGluT1 (**Figure 2A**), cone photoreceptors were visualized using retinae of Rac3-EGFP mice, which express eGFP in their cone photoreceptors (**Figure 2B**), and ON bipolar cells were detected using retinae of Lrrc55-EGFP mice, which express eGFP in their ON bipolar cells (**Figure 2C**; Regus-Leidig et al., 2013). EGFP of transgenic retinae was intensified with an antibody against GFP.

The majority of analyzed endocytotic components was present and diffusely distributed in all three types of ribbon synapses, i.e., in terminals of rod (**Figure 2D**) and cone (**Figure 2E**) photoreceptors, and in ON bipolar cell terminals (**Figure 2F**). Synaptojanin1 was the only protein which was almost undetectable in light adapted rod and cone photoreceptor terminals (**Figures 2D,E**); in ON bipolar cell terminals it was diffusely distributed (**Figure 2F**). Although some of these proteins might also be involved in other modes of endocytosis (Nichols and Lippincott-Schwartz, 2001; Jockusch et al., 2005; Llobet et al., 2011), from our findings we conclude that CME is an important contributor to endocytosis at retinal ribbon synapses with only little variation between photoreceptors and ON bipolar cells regarding the analyzed protein variants. However, immunolabeling for AP180 and Clathrin LC appeared weaker in cone terminals than in rod terminals (**Figures 2D,E**). This hints at possible rod-cone differences in the expression of these proteins and thus may distinguish the two photoreceptor types’ dependence on CME for synaptic vesicle retrieval. Evidence that endocytosis in salamander rods and cones involves different mechanisms has recently been provided by Van Hook and Thoreson (2012).

### ULTRASTRUCTURAL ANALYSIS OF CME IN PHOTORECEPTOR RIBBON SYNAPSES

**FIGURE 3:**
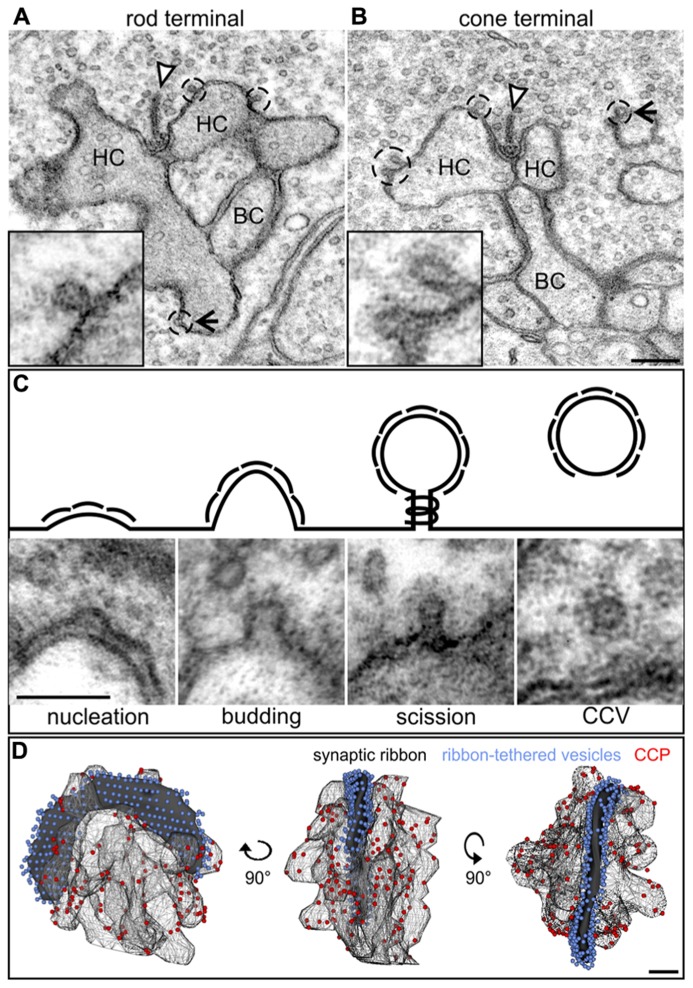
**Ultrastructural analysis of Clathrin-mediated endocytosis in photoreceptor ribbon synapses in a 3 h light adapted retina. (A,B)** Representative electron micrographs of a rod **(A)** and cone photoreceptor **(B)** terminal in the light adapted mouse retina. Clathrin-coated pits (CCP) budding off from the plasma membrane are demarcated with dashed lines and magnified in the insets. Arrowheads point to synaptic ribbons; arrows to CCP remote from synaptic ribbons. **(C)** Exemplary electron micrographs of Clathrin-coated structures from the different stages of Clathrin-mediated endocytosis (schematized above) in rod photoreceptor terminals. **(D)** 3D-reconstruction of a light adapted rod photoreceptor terminal viewed from different angles. The horseshoe-shaped synaptic ribbon (black) bends around the postsynaptic invaginations of bipolar and horizontal cell processes. Ribbon-tethered vesicles are shown in blue, CCP are shown in red. HC, horizontal cell; BC, bipolar cell. Scale bar in **B** (for **A,B**): 200 nm, in **C**: 100 nm, in **D**: 200 nm.

In contrast to the diffuse distribution of endocytotic proteins in our light microscopical analysis (**Figure 2**), a ring-shaped concentration of Dynamin around the rod photoreceptor synaptic ribbon corresponding to a circular hotspot of endocytosis has been reported (Wahl et al., 2013). To analyze the distribution of CCP in relation to photoreceptor synaptic ribbons in light adapted retinae, we performed an ultrastructural analysis of rod and cone photoreceptor ribbon synapses in the BL/6 retina (**Figure 3**). CCP (**Figures 3A,B**; dashed circles and high power view insets) were found in the periactive zones at various distances from the synaptic ribbons (arrowhead) in both rod and cones. To better judge the distribution of CCP in the periactive zone, we 3D-reconstructed rod photoreceptor synaptic terminals (*n* = 3) from serial ultrathin sections. For reconstruction we did not differentiate between the different stages of CCP, and reconstructed all Clathrin-coated structures from the nucleation stage to the scission stage (**Figure 3C**). **Figure 3D** shows a representative example of a light adapted rod photoreceptor ribbon synaptic complex viewed from three different angles rotated by 90°: synaptic ribbon (black), tethered synaptic vesicles (blue spheres), invaginating postsynaptic elements (Boissonnat surfaces), and CCP (red spheres). CCP were frequently observed in the periactive zone, but never directly at the base of the synaptic ribbon. They appeared not to be enriched in a ring-shaped distribution around the synaptic ribbon, but were distributed all-over the plasma membrane of the invaginating postsynaptic elements (**Figure 3D**). Free CCV could rarely be seen in the cytoplasm of rod photoreceptor terminals (**Figure 3C**), most likely indicating rapid uncoating after scission (Brodin et al., 2000).

### ENDOCYTOTIC PROTEINS REDISTRIBUTE IN PHOTORECEPTOR TERMINALS FOLLOWING DARK EXPOSURE

The reported enrichment of Dynamin in the periactive zone surrounding the photoreceptor synaptic ribbon complex (Wahl et al., 2013) is at odds both with our light microscopical finding of a diffuse distribution of endocytotic proteins including Dynamin3 (**Figures 2D,E**), and with the ultrastructural distribution of CCP in our 3D-reconstructed rod photoreceptor ribbon synaptic complexes (**Figure 3D**). The different anti-Dynamin antibodies used in the study by Wahl et al. (2013) and our study are not the reason for the differing results. Wahl et al. (2013) used a monoclonal mouse anti-Dynamin1 antibody generated against a peptide stretch in the C-terminal region of Dynamin1 that is also conserved in Dynamin2 and Dynamin3, thus most likely not distinguishing between the three Dynamin forms. In our study, we used two anti-Dynamin antibodies recognizing all three Dynamin forms and an anti-Dynamin3 specific antibody (Section Materials and Methods). The pan Dynamin antibodies and the anti-Dynamin3 antibody produced the same staining pattern in the immunocytochemical experiments, suggesting that Dynamin3 is the major Dynamin form at photoreceptor synaptic terminals.

**FIGURE 4:**
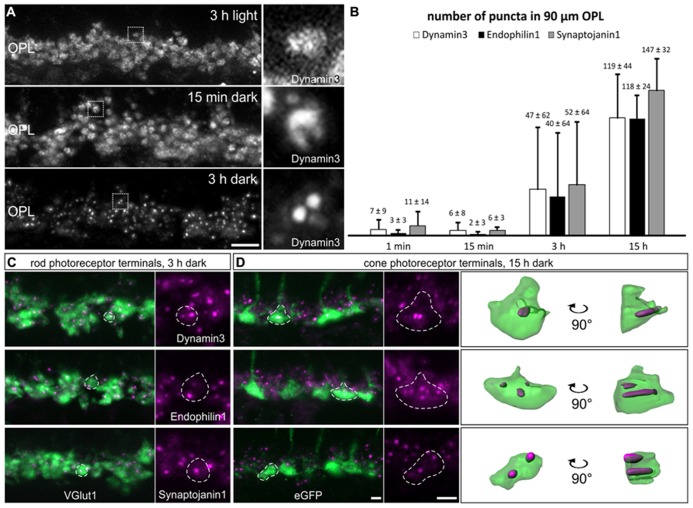
**Redistribution of endocytotic proteins in 1 min, 15 min, 3 h, and 15 h dark adapted photoreceptor terminals. (A)** Outer plexiform layer (OPL) of C57BL/6JRj retinae after different periods of dark adaptation labeled with an antibody against Dynamin3. Single synaptic terminals are shown in high magnification. **(B)** Quantification of immunofluorescent Dynamin3-, Endophilin1-, and Synaptojanin1 puncta in the OPL after different durations of dark adaptation. **(C)** Images of rod photoreceptor terminals of C57BL/6JRj retina after 3 h of dark adaptation stained with antibodies against VGluT1 (green) and Dynamin3, Endophilin1, or Synaptojanin1 (magenta). **(D)** Images of cone photoreceptor terminals of Rac3-EGFP retina after 15 h of dark adaptation stained with antibodies against GFP (green) and Dynamin3, Endophilin1, or Synaptojanin1 (magenta). Single synaptic terminals are delineated with dashed lines. Values in **B** are means ± SD (*n* = 4 retinae from 4 mice, 3 images per retina). Scale bar in **A**: 10 μm, in **D** (for **C,D**): 2 μm.

To figure out what might be the reason for the discrepancy between our study and the study by Wahl et al. (2013), and since all our experiments so far had been performed on light adapted retinae, we examined the localization of endocytotic proteins following increasing periods of dark exposure, i.e., increasing synaptic activity. Mice were light adapted for 3 h and then dark adapted for 1 min, 15 min, 3 h, and 15 h. As shown in **Figure 4A**, Dynamin3 underwent a remarkable redistribution in the OPL in the dark adapted retinae; note that no obvious adaptation-dependent differences in Dynamin3 distribution were detected in the terminals of ON bipolar cells at any time point (data not shown). Already after 1 to 15 min of increased activity, Dynamin3 labeling changed from a diffuse to a circular appearance (**Figure 4A**), which is in line with the Dynamin distribution reported by Wahl et al. (2013). The same staining patterns after 1 and 15 min of darkness were observed for Endophilin1, Amphiphysin1, Clathrin LC, and AP180 (data not shown), reflecting a clustering of endocytotic proteins in the periactive zone during periods of increased exocytosis.

The most striking redistribution of Dynamin3 was seen after prolonged periods of darkness (3 to 15 h), when distinct Dynamin3 hotspots appeared throughout the entire OPL in the rod and cone photoreceptor synaptic terminals (**Figures 4A,C,D**). An antibody against VGluT1 was used to label both rod and cone photoreceptor terminals (**Figure 4C**). To be able to distinguish between rod and cone photoreceptor terminals, we additionally performed the stainings on Rac3-EGFP retinae which, express eGFP in the cone photoreceptor terminals (**Figure 4D**). Endophilin1, and most interestingly also Synaptojanin1 (**Figures 4C,D**) which had been virtually undetectable in the OPL in the light adapted retinae (**Figures 2D,E**) redistributed in the photoreceptor synaptic terminals similar to Dynamin3 after prolonged dark exposure. For Amphiphysin1 scattered immunofluorescent puncta could be detected, whereas Clathrin LC, AP180, and hsc70 were never observed to aggregate after prolonged dark periods (data not shown). The quantification of Dynamin3, Endophilin1, and Synaptojanin1 puncta in stretches of 90 μm OPL after the different durations of dark exposure is shown in **Figure 4B**. After 1 and 15 min of dark adaptation, immunofluorescent puncta for Dynamin3, Endophilin1, and Synaptojanin1 were rarely found, while the number of puncta markedly increased for all three proteins after 3 and 15 h of dark adaptation (**Figure 4B**). After 3 h of dark adaptation, almost all rod photoreceptor terminals contained one or two Dynamin3, Endophilin1, and Synaptojanin1 immunofluorescent puncta (**Figure 4C**). In the cone photoreceptor terminals, a punctate staining of the three endocytotic proteins was more frequent after 15 h of dark adaptation, with a higher number of puncta per terminal than in rod photoreceptor terminals (**Figure 4D**). 3D reconstructions confirmed the localization of immunofluorescent puncta within the cone photoreceptor terminals (**Figure 4D**).

### PUNCTATE COLOCALIZATION OF DYNAMIN3, ENDOPHILIN1, AND SYNAPTOJANIN1 IN PHOTORECEPTOR SYNAPTIC TERMINALS AFTER PROLONGED DARK EXPOSURE

**Figure 5:**
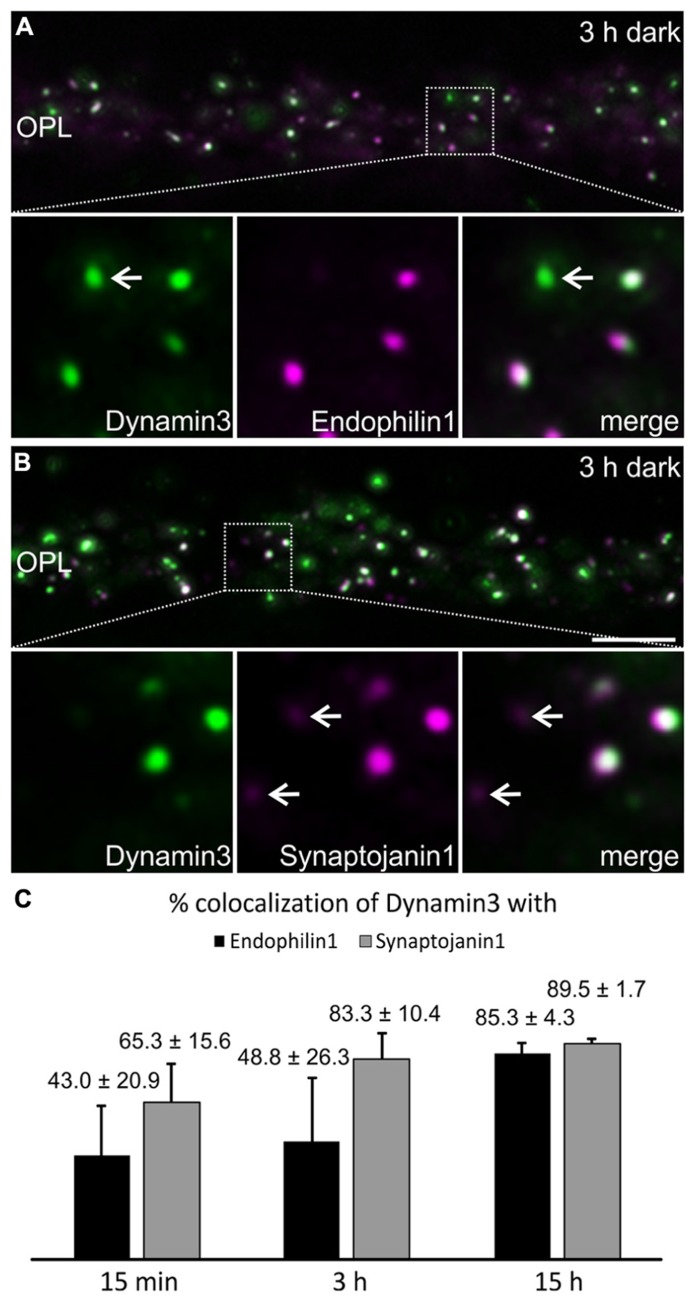
**Colocalization of Dynamin3 with Endophilin1 and Synaptojanin1 in 15 min, 3 h, and 15 h dark adapted OPL. (A,B)** Outer plexiform layer (OPL) of C57BL/6JRj retinae after 3 h of dark adaptation labeled with antibodies against Dynamin3 (green) and Endophilin1 **(A)** or Synaptojanin1 (**B**; magenta). Arrows in the high power views point to individual, non-colocalizing puncta. **(C)** Quantification of the colocalization of Dynamin3 puncta with Endophilin1 and Synaptojanin1 in the OPL after 15 min, 3 h, and 15 h of dark adaptation. Values are means ± SD (*n* = 3 retinae from 3 mice, 3 images per retina). Scale bar in **B** (for **A,B**): 5 μm.

Dynamin3, Endophilin1, and Synaptojanin1 displayed a punctate staining in the photoreceptor synaptic terminals after prolonged dark exposure (**Figure 4**). To test for colocalization of the three endocytotic proteins, we performed double labeling experiments for Dynamin3/Endophilin1, and Dynamin3/Synaptojanin1 (**Figures 5A,B**). Colocalization of Dynamin3 with Endophilin1 and Synaptojanin1, respectively, was apparent, although not complete, since single Dynamin3-, Endophilin1-, and Synaptojanin1-positive puncta were detected as well (**Figures 5A,B**; arrows). The percentage of Dynamin3 puncta that colocalized with Endophilin1 and Synaptojanin1 puncta increased with increasing periods of dark exposure (**Figure 5C**). At 15 h dark adaptation there was almost complete colocalization of the three endocytotic proteins (**Figure 5C**).

### ULTRASTRUCTURAL CHARACTERIZATION OF AN ENDOCYTOTIC VESICLE CLUSTER IN DARK ADAPTED PHOTORECEPTOR TERMINALS

**FIGURE 6:**
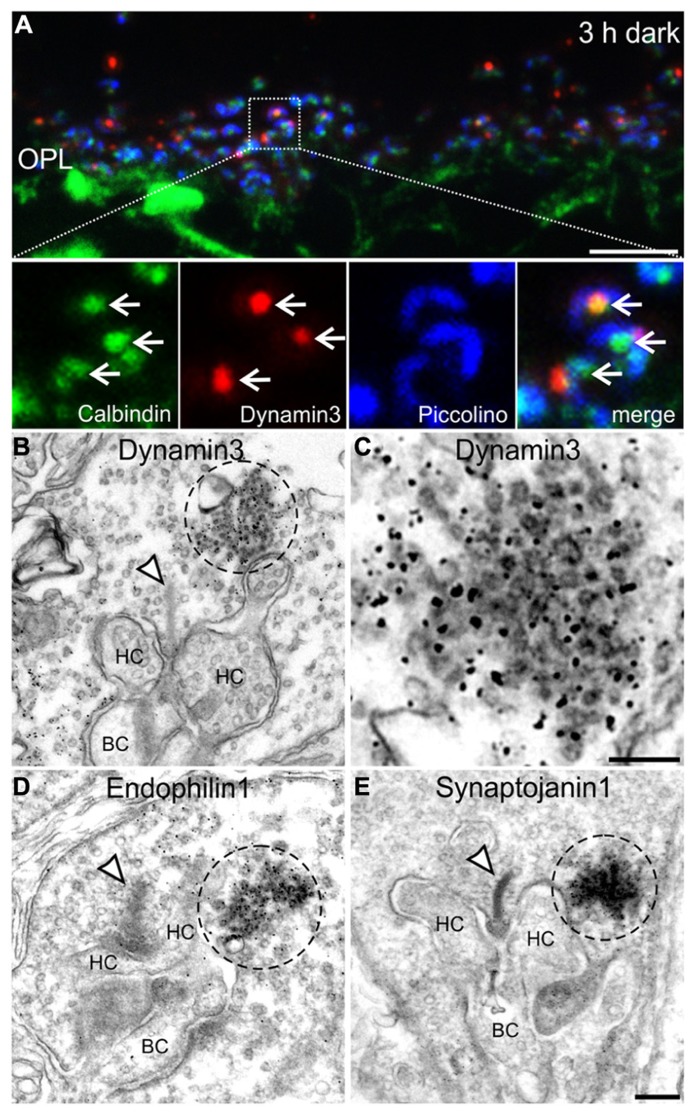
**Presynaptic localization of Dynamin3, Endophilin1, and Synaptojanin1 in 3 h dark adapted rod photoreceptor terminals. (A)** Outer plexiform layer (OPL) of C57BL/6JRj retina after 3 h of dark adaptation labeled with antibodies against Calbindin (green), Dynamin3 (red), and Piccolino (blue). Arrows point to adjacent Calbindin- and Dynamin3-puncta. **(B–E)** Pre-embedding immunoelectron micrographs of 3 h dark adapted rod photoreceptor terminals stained with antibodies against Dynamin3 **(B,C)**, Endophilin1 **(D)**, and Synaptojanin1 **(E)**. Immunoreactivity is encircled with dashed lines; arrowheads point to synaptic ribbons. HC, horizontal cell; BC, bipolar cell. Scale bar in **A**: 5 μm, in **D** (for **B,D,E**): 200 nm, in **C**: 100 nm.

**FIGURE 7:**
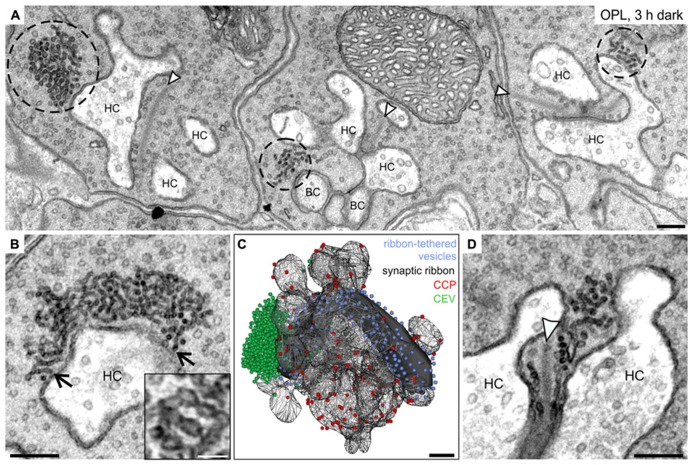
**Ultrastructural analysis of clusters of electron-dense endocytotic vesicles in 3 h dark adapted rod photoreceptor terminals. (A)** Electron micrograph of outer plexiform layer (OPL), showing clusters of electron dense endocytotic vesicles (CEV; encircled with dashed lines) in three neighboring rod photoreceptor terminals of a 3 h dark adapted C57BL/6JRj retina. **(B)** Representative electron micrograph of a rod photoreceptor terminal in the 3 h dark adapted retina. Arrows point to single vesicles of the CEV emanating from the plasma membrane at the postsynaptic invaginations. The high magnification inset shows the membranous connection between individual vesicles of a CEV. **(C) **3D-reconstruction of a 3 h dark adapted rod photoreceptor terminal. The horseshoe-shaped synaptic ribbon (black) bends around the postsynaptic invaginations of bipolar and horizontal cells. Ribbon-tethered vesicles are shown in blue, Clathrin-coated pits (CCP) in red, and CEV in green. **(D)** Electron micrograph of a 3 h dark adapted rod photoreceptor terminal showing CEV in close association with vesicles tethered to the synaptic ribbon. Arrowheads point to synaptic ribbons. HC, horizontal cell; BC, bipolar cell. Scale bar in **A–D**: 200 nm, inset in **B**: 50 nm.

To analyze the presynaptic localization of the punctate immunoreactivity for Dynamin3, Endophilin1, and Synaptojanin1, we first triple labeled cryostat sections of 3 h dark adapted BL/6 retinae for Calbindin (green; labels horizontal cells and their invaginations into photoreceptor terminals), Dynamin3 (red), and Piccolino, a ribbon-specific Piccolo variant labeling the synaptic ribbon (blue; Regus-Leidig et al., 2013; **Figure 6A**). The merge of the three stainings shows that Dynamin3 puncta localize in the periactive zone close to the horseshoe shaped synaptic ribbons as well as the tips of horizontal cell processes (**Figure 6A**; arrows). To characterize this putative endocytotic hot spot at the ultrastructural level, we next performed pre-embedding immunoelectron microscopy on 3 h dark adapted BL/6 retinae with antibodies against Dynamin3, Endophilin1, Synaptojanin1 (**Figures 6B–E**), and Clathrin (data not shown). We also tried post-embedding immunoelectron microscopy, which unfortunately did not satisfactorily work in our hands. In rod photoreceptor terminals, immunoreactivity for Dynamin3, Endophilin1, Synaptojanin1 concentrated in the vicinity of the postsynaptic invaginations and the synaptic ribbon (**Figures 6B–E**; dashed circles). As expected, Clathrin LC immunoreactivity was diffusely distributed throughout the photoreceptor synaptic terminals, confirming the light microscopical absence of Clathrin LC in these endocytotic hot spots (data not shown). Sometimes, presumptive vesicles were visible in the labeled region (**Figure 6C**), but most of the times the intense staining obscured any underlying structures. Therefore, we analyzed dark adapted BL/6 retinae with conventional electron microscopy for the presence of conspicuous vesicle clusters in the vicinity of the synaptic ribbon (**Figure 7**).

While we could not detect any obvious ultrastructural changes in rod and cone photoreceptor terminals comparing light and shortly dark exposed retinae (1 and 15 min), we observed striking accumulations of electron-dense vesicles (dashed circles) in the vicinity of synaptic ribbons (arrowheads) and the postsynaptic elements after 3 h, and 15 h (data not shown) of dark exposure in rod photoreceptor terminals (**Figure 7A**). These electron-dense vesicles could also be observed in cone terminals, albeit less frequently. Occasionally, these vesicle clusters were seen to emanate from the plasma membrane at the rod postsynaptic invaginations suggesting an endocytotic origin (**Figure 7B**; arrows). The closely connected vesicles in the cluster (**Figure 7B**; inset) were smaller than synaptic vesicles (mean diameter of electron-dense vesicles: 28.8 ± 5.2 nm, SD, *n* = 1100; mean diameter of synaptic vesicles: 37.3 ± 3.8 nm, SD, *n* = 1100). 3D reconstructions of prolonged dark adapted rod photoreceptor terminals revealed the great extent of the clusters of electron-dense endocytotic vesicles (CEV; green; **Figure 7C**) and confirmed their localization at the periactive zone and their connection to the plasma membrane. The vesicle numbers in 3D reconstructed CEV from seven photoreceptor terminals after 3 h of dark adaptation ranged from 291 to 1371 vesicles (mean: 695 ± 381, SD). CEV were also present in photoreceptor terminals of dark adapted Balb/c mice and Dark Agouti rats (data not shown), excluding a strain or species specific phenomenon. Of note, we never observed CEV in ON bipolar cell terminals of light or dark adapted retinae, corroborating the finding that Dynamin3 did not redistribute in the ON bipolar cell terminals following light or dark adaptation.

Finally, the number of CCP determined from single ultrathin sections (**Figure 7C**; red spheres) increased with increasing periods of dark exposure, reaching highest numbers in the 15 h dark adapted state: light: 8.4 ± 2.8; 1 min dark: 7.7 ± 2.7; 15 min dark: 13.2 ± 2.9; 3 h dark: 13.9 ± 4.1; 15 h dark: 15.0 ± 3.9; SD (*n* = 50 terminals per retina; three retinae from three animals per adaptation state). It is important to note that, comparable to the light adapted state (**Figure 3**), also in the prolonged dark adapted retinae, CCP were not enriched around the ribbon synaptic site but evenly distributed along the plasma membrane of the postsynaptic complex (**Figure 7C**).

### CEV FORMATION IN PHOTORECEPTOR TERMINALS IN THE ABSENCE OF FUNCTIONAL BASSOON

**Figure 8:**
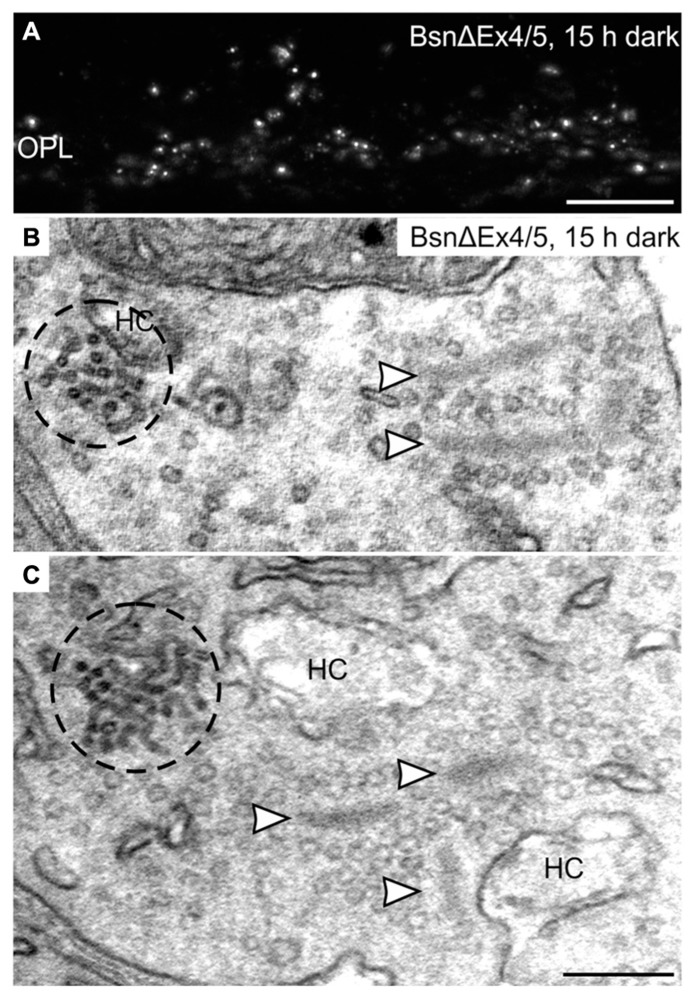
**Clusters of electron-dense endocytotic vesicles in 15 h dark adapted BsnΔEx4/5 photoreceptor terminals. (A) **Outer plexiform layer (OPL) of a 15 h dark adapted BsnΔEx4/5 retina labeled with an antibody against Dynamin3. **(B,C)** Electron micrographs of rod photoreceptor terminals in a 15 h dark adapted BsnΔEx4/5 retina. Clusters of electron dense endocytotic vesicles (CEV) are encircled with dashed lines; arrowheads point to synaptic ribbons. HC, horizontal cell. Scale bar in **A**: 10 μm, in **C** (for **B,C**): 200 nm.

Clusters of electron-dense endocytotic vesicles were sometimes seen in close association with vesicles at the synaptic ribbon (**Figure 7D**; arrowhead), hinting at a contribution of the synaptic ribbon to vesicle retrieval as postulated previously (Schmitz, 2009; Schwarz et al., 2011; Wahl et al., 2013). To analyze the importance of the synaptic ribbon for the formation of CEV, we made use of the Bassoon mutant mouse (BsnΔEx4/5), which possesses detached, free floating photoreceptor synaptic ribbons due to a missing interaction of the anchor molecule Bassoon with RIBEYE (Dick et al., 2003; tom Dieck et al., 2005; Regus-Leidig et al., 2010b). Despite the absence of active zone anchored ribbons, punctate hotspots of Dynamin3 immunoreactivity were present in the photoreceptor terminals of the Bassoon mutant mouse after prolonged dark exposure (**Figure 8A**). Electron microscopy confirms the presence of CEV and, most importantly, the clear spatial separation of CEV and free ribbons the Bassoon mutant photoreceptor terminals (**Figures 8B,C**).

## DISCUSSION

**FIGURE 9:**
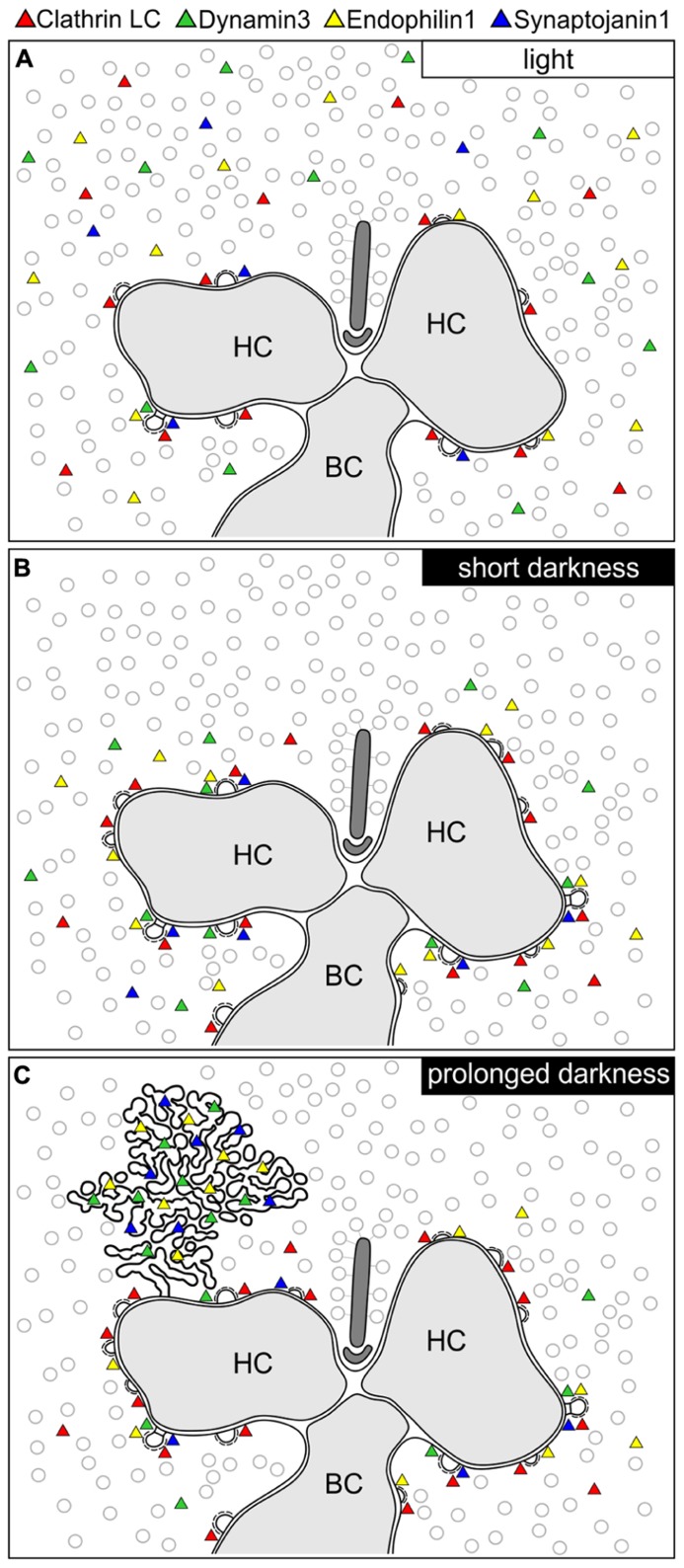
**Schematic summary of the hypothesized light/activity-dependent modes of endocytosis in photoreceptor terminals. (A–C)** Photoreceptor ribbon synaptic site showing the postulated modes of endocytosis and the distribution of Dynamin3, Endophilin1, Synaptojanin1, and Clathrin LC (represented as colored triangles) in the light adapted state **(A)**, after short periods of darkness (1 to 15 min; **B**), and after prolonged periods of darkness (3 to 15 h; **C**). HC, horizontal cell; BC, bipolar cell.

In the present study, we analyzed the presynaptic localization of the endocytotic proteins Clathrin LC, AP180, Endophilin1, Amphiphysin1, Synaptojanin1, Dynamin3, and hsc70 at retinal ribbon synapses as a function of synaptic activity (light/dark). We found an adaptation- and thus activity-dependent redistribution of Dynamin3, Endophilin1, and Synaptojanin1 in rod and cone photoreceptor ribbon synapses. **Figure 9** summarizes the main results of our study:

### LIGHT/LOW PHOTORECEPTOR SYNAPTIC ACTIVITY

In light adapted photoreceptor synapses, CME is the predominant mode of endocytosis. Due to low rates of endocytosis in the periactive zone, the majority of Clathrin LC, Dynamin3, Endophilin1, and Synaptojanin1 was diffusely distributed in the cytoplasm (**Figure 9A**). In line with minimal synaptic activity, we found the lowest number of CCP in photoreceptor synaptic terminals of light adapted retinae.

### SHORT DARKNESS/HIGH PHOTORECEPTOR SYNAPTIC ACTIVITY (1 TO 15 min)

In shortly dark exposed photoreceptor ribbon synapses, Clathrin LC, Dynamin3, Endophilin1, and Synaptojanin1 concentrated in the periactive zone surrounding the photoreceptor synaptic ribbon complex. This most likely reflects increased membrane turnover rates in conjunction with increased synaptic activity, a finding which is in agreement with the concept of periactive zone endocytosis in photoreceptor ribbon synapses (Wahl et al., 2013). In line with the assumption of high sustained photoreceptor synaptic activity in darkness, the number of CCP increased in the photoreceptor terminals following dark exposure.

### PROLONGED DARKNESS/HIGH PHOTORECEPTOR SYNAPTIC ACTIVITY (3 TO 15 h)

After prolonged dark exposure, the number of CCP in the photoreceptor terminals was highest, and an additional mode of endocytosis involving Dynamin3, Endophilin1, and Synaptojanin1 occurred. The latter resulted in the uptake of membranously connected, electron-dense endocytotic vesicles (CEV; **Figure 9C**).

Both types of photoreceptors showed similar activity-dependent protein redistribution and ultrastructure of CEV. Differences in temporal appearance (CEV in rod photoreceptor terminals occured earlier than in cone photoreceptor terminals) and number of CEV per terminal (one or two CEV in rod photoreceptor terminals, more than two CEV in cone photoreceptor terminals) possibly reflect the different functional demands on rod and cone photoreceptors.

Clathrin LC, AP180, and hsc70 are involved in the formation and degradation of the Clathrin coat (for review, see Edeling et al., 2006; McMahon and Boucrot, 2011). The absence of these proteins in the CEV, together with the ultrastructural lack of Clathrin coats associated with the CEV, corroborate a Clathrin-independent formation of these endocytotic vesicles, and suggest that photoreceptor ribbon synapses possess, in addition to CME, a Clathrin-independent mode of vesicle retrieval initiated during prolonged high synaptic activity. We could also show that the formation of CEV is not dependent on the presence of anchored synaptic ribbons, as CEV also formed in the BsnΔEx4/5 mouse retina, in which photoreceptor synaptic ribbons are not membrane-anchored but free floating in the synaptic terminals.

Wahl et al. (2013) reported punctate staining in rod photoreceptor terminals with an antibody against Clathrin heavy chain (HC), resembling the punctate staining we observed for Dynamin3, Endophilin1, and Synaptojanin1 after prolonged dark exposure. However, these puncta do not correspond to the CEV shown in our study. First, the Clathrin HC puncta were found in light adapted photoreceptors, and second, they were located a large distance (~580 nm) away from the synaptic ribbon. Furthermore, the authors interpret the Clathrin HC puncta to represent an endosomal compartment in the proximal region of the photoreceptor terminal (Wahl et al., 2013).

Organelles resembling the CEV have been described before in photoreceptors from various species as “tubular complexes/profiles/networks,” “branched tubular structures,” “accumulations of vesicle-like particles,” “tubulovesicular structures,” or “endoplasmatic reticulum (ER)-like structures” (Yamada, 1965; Samorajski, 1966; Lovas, 1971; Babai et al., 2010; Koch et al., 2011; López-del Hoyo et al., 2012). López-del Hoyo et al. (2012) hypothesized that these aggregates might be generated as “by-products in the bulk endocytosis,” but no functional correlation with the level of synaptic activity was made. We are confident that the vesicle clusters we describe here are of endocytotic origin and do not represent ER for three reasons: (1) The clusters appear only after prolonged dark exposure, (2) they are connected to the presynaptic plasma membrane, and (3) they were never seen to be connected to other intracellular membrane structures.

The temporal appearance of CEV in dark adapted mouse photoreceptor terminals did not follow any general rule and was not directly correlated with the duration of dark exposure (see high standard deviation in **Figure 4B**). So far, we can therefore only speculate about the trigger and functional significance of CEV formation. At central nerve terminals, activity-dependent bulk endocytosis as a second vesicle retrieval mode has been reported in reaction to increased stimulation (Clayton et al., 2008; Dittman and Ryan, 2009), and the phosphorylation of Dynamin was shown to act as a switch from CME to additional bulk endocytosis (Clayton et al., 2009). The presence of CEV in photoreceptor terminals might therefore represent bulk endocytosis after prolonged high synaptic activity. However, bulk endocytosis, as also reported for bipolar cell ribbon synapses of the goldfish retina, involves the uptake of large bites of membrane with subsequent generation of synaptic vesicles (LoGiudice and Matthews, 2007). To our knowledge, bulk endocytosis consisting of individual, membranously connected vesicles has never been reported so far. CEV might represent a form of bulk vesicle retrieval without detour via endosomal intermediates for the rapid formation of releasable vesicles in continuously active photoreceptors, especially during sustained maximal activity (Rea et al., 2004). Although we sometimes found vesicles and/or tubules of CEV in close vicinity to synaptic ribbons, we doubt that CEV provide a direct reloading of the synaptic ribbon with new synaptic vesicles. In the majority of ultrathin sections as well as in all 3D reconstructions from rod terminals, CEV were localized at the presynaptic plasma membrane some distance away from the synaptic ribbon.

Curiously, although Dynamin as the required mechano-enzyme for the final fission step in vesicle formation is present on CEV, CEV are not accumulations of free vesicles but aggregates of membranously connected vesicles. In the absence of functional Dynamin1 (and 3) similar branched tubular networks, albeit with Clathrin-coats, were detected at mouse conventional chemical synapses (Ferguson et al., 2007; Raimondi et al., 2011). Therefore, instead of being a high-throughput endocytotic hotspot during sustained maximal activity, CEV might arise as an artifact during impaired vesicle retrieval, e.g., due to a limited availability of essential proteins or other factors during excessive synaptic activity. Since the GTPase Dynamin requires energy for its fission reaction (Ferguson and de Camilli, 2012), it is conceivable that accumulations of connected endocytotic vesicles might result during periods of energy shortage in the photoreceptor synaptic terminal. Since many steps in the presynapse are energy-consuming, e.g., priming of synaptic vesicles, the regulation of the [Ca^2^^+^]_i_ by the plasma membrane Calcium-ATPase, and acidification of synaptic vesicles by the vacuolar H^+^-ATPase (reviewed in Südhof and Rizo, 2011; Harris et al., 2012), prolonged high synaptic activity may lead to metabolic stress in this cellular compartment.

However, although the impairment of compensatory endocytosis in conventional chemical synapses in the absence of Dynamin strongly impacts the availability of synaptic vesicles for exocytosis (Ferguson et al., 2007; Raimondi et al., 2011), we doubt that CEV as a putative sign of arrest in vesicle recycling would have any perturbing effect on photoreceptor synaptic transmission. The percentage of “retained” vesicles in a CEV in relation to the number of free synaptic vesicles in the presynaptic cytoplasm is less than 10% (judged from the vesicle numbers counted in serial sections of three 3D reconstructed dark adapted rod photoreceptor terminals), and thus would hardly limit the availability of releasable vesicles.

As in the photoreceptor terminals, also in the ON bipolar cell terminals we detected all analyzed endocytotic proteins, which is in line with the study by LoGiudice et al. (2009), who reported a Clathrin-mediated pathway for the retrieval of synaptic vesicles in mouse bipolar cell terminals. In contrast to the changing labeling pattern in photoreceptor terminals, the endocytotic proteins showed a diffuse distribution in bipolar cell terminals at all evaluated conditions. Moreover, at the ultrastructural level we never found endocytotic structures other than CCP, indicating CME as a major mode of endocytosis in mouse ON bipolar cell synapses in various activity states. The absence of CEV as putative membrane retrieval mechanism under prolonged activity may reflect the lower membrane turnover in bipolar cell terminals compared to photoreceptors synapses.

In conclusion, from our analysis of retinae in different adaptational states, we propose that the occurrence of CEV in the photoreceptor terminal reflects a stage of high synaptic activity. In future studies, it remains to be determined if CEV represent a special adaptation to the photoreceptors’ need for continuous vesicle supply during high activity or rather a pathological artifact as a result of excessive stress.

## AUTHOR CONTRIBUTIONS

Hanna Regus-Leidig and Michaela Fuchs designed research, conducted research, and analyzed data. Hanna Regus-Leidig, Michaela Fuchs and Johann Helmut Brandstätter wrote the paper.

## Conflict of Interest Statement

The authors declare that the research was conducted in the absence of any commercial or financial relationships that could be construed as a potential conflict of interest.

## References

[B1] Altrock W. D. tom Dieck S. Sokolov M. Meyer A. C. Sigler A. Brakebusch C. (2003). Functional inactivation of a fraction of excitatory synapses in mice deficient for the active zone protein bassoon. *Neuron* 37 787–800 10.1016/S0896-6273(03)00088-6 12628169

[B2] Babai N. Morgans C. W. Thoreson W. B. (2010). Calcium-induced calcium release contributes to synaptic release from mouse rod photoreceptors. *Neuroscience* 165 1447–1456 10.1016/j.neuroscience.2009.11.032 PMC2815208 19932743

[B3] Bai L. Spiwoks-Becker I. Leube R. E. (2006). Transcriptome comparison of murine wild-type and synaptophysin-deficient retina reveals complete identity. *Brain Res.* 1081 53–58 10.1016/j.brainres.2006.01.080 16519878

[B4] Brandstätter J. H. Löhrke S. Morgans C. W Wässle H. (1996). Distributions of two homologous synaptic vesicle proteins, synaptoporin and synaptophysin, in the mammalian retina. *J. Comp. Neurol.* 370 1–10 10.1002/(SICI)1096-9861(19960617)370:1<1::AID-CNE1>3.0.CO;2-7 8797152

[B5] Brodin L. Löw P. Shupliakov O. (2000). Sequential steps in clathrin-mediated synaptic vesicle endocytosis. *Curr. Opin. Neurobiol.* 10 312–320 10.1016/S0959-4388(00)00097-0 10851177

[B6] Clayton E. L. Anggono V. Smillie K. J. Chau N. Robinson P. J. Cousin M. A. (2009). The phospho-dependent dynamin-syndapin interaction triggers activity-dependent bulk endocytosis of synaptic vesicles. *J. Neurosci.* 29 7706–7717 10.1523/JNEUROSCI.1976-09.2009 PMC2713864 19535582

[B7] Clayton E. L. Evans G. J. O. Cousin M. A. (2008). Bulk synaptic vesicle endocytosis is rapidly triggered during strong stimulation. *J. Neurosci.* 28 6627–6632 10.1523/JNEUROSCI.1445-08.2008 PMC2588494 18579735

[B8] Dick O. Hack I. Altrock W. D. Garner C. C. Gundelfinger E. D Brandstätter J. H. (2001). Localization of the presynaptic cytomatrix protein Piccolo at ribbon and conventional synapses in the rat retina: comparison with Bassoon. *J. Comp. Neurol.* 439 224–234 10.1002/cne.1344 11596050

[B9] Dick O. tom Dieck S. Altrock W. D. Ammermüller J. Weiler R. Garner C. C. (2003). The presynaptic active zone protein bassoon is essential for photoreceptor ribbon synapse formation in the retina. *Neuron* 37 775–786 10.1016/S0896-6273(03)00086-2 12628168

[B10] Dittman J. Ryan T. A. (2009). Molecular circuitry of endocytosis at nerve terminals. *Annu. Rev. Cell Dev. Biol.* 25 133–160 10.1146/annurev.cellbio.042308.113302 19575674

[B11] Edeling M. A. Smith C. Owen D. (2006). Life of a clathrin coat: insights from clathrin and AP structures. *Nat. Rev. Mol. Cell Biol.* 7 32–44 10.1038/nrm1786 16493411

[B12] Ferguson S. M. Brasnjo G. Hayashi M. Wölfel M. Collesi C. Giovedi S. (2007). A selective activity-dependent requirement for dynamin 1 in synaptic vesicle endocytosis. *Science* 316 570–574 10.1126/science.1140621 17463283

[B13] Ferguson S. M de Camilli P. (2012). Dynamin, a membrane-remodelling GTPase. *Nat. Rev. Mol. Cell Biol.* 13 75–88 10.1038/nrm3266 PMC3519936 22233676

[B14] Gray E. G. Pease H. L. (1971). On understanding the organisation of the retinal receptor synapses. *Brain Res.* 35 1–15 10.1016/0006-8993(71)90591-9 5134225

[B15] Grossman G. H. Ebke L. A. Beight C. D. Jang G.-F. Crabb J. W. Hagstrom S. A. (2013). Protein partners of dynamin-1 in the retina. *Vis. Neurosci.* 30 129–139 10.1017/S0952523813000138 PMC3936680 23746204

[B16] Harris J. J. Jolivet R. Attwell D. (2012). Synaptic energy use and supply. *Neuron* 75 762–777 10.1016/j.neuron.2012.08.019 22958818

[B17] Haucke V. Neher E. Sigrist S. J. (2011). Protein scaffolds in the coupling of synaptic exocytosis and endocytosis. *Nat. Rev. Neurosci.* 12 127–138 10.1038/nrn2948 21304549

[B18] Heidelberger R. Thoreson W. B. Witkovsky P. (2005). Synaptic transmission at retinal ribbon synapses. *Prog. Retin. Eye Res.* 24 682–720 10.1016/j.preteyeres.2005.04.002 16027025 PMC1383430

[B19] Holzhausen L. C. Lewis A. A. Cheong K. K. Brockerhoff S. E. (2009). Differential role for synaptojanin 1 in rod and cone photoreceptors. *J. Comp. Neurol.* 517 633–644 10.1002/cne.22176 PMC3071606 19827152

[B20] Jockusch W. J. Praefcke G. J. McMahon H. T. Lagnado L. (2005). Clathrin-dependent and clathrin-independent retrieval of synaptic vesicles in retinal bipolar cells. *Neuron* 46 869–878 10.1016/j.neuron.2005.05.004 15953416

[B21] Kachi S. Yamazaki A. Usukura J. (2001). Localization of caveolin-1 in photoreceptor synaptic ribbons. *Invest. Ophthalmol. Vis. Sci.* 42 850–852 11222549

[B22] Kim H. Lee T. Lee J. Ahn M. Moon C. Wie M. B. (2006). Immunohistochemical study of caveolin-1 and -2 in the rat retina. *J. Vet. Sci.* 7 101–104 10.4142/jvs.2006.7.2.101 16645331 PMC3242098

[B23] Koch D. Spiwoks-Becker I. Sabanov V. Sinning A. Dugladze T. Stellmacher A. (2011). Proper synaptic vesicle formation and neuronal network activity critically rely on syndapin I. *EMBO J.* 30 4955–4969 10.1038/emboj.2011.339 PMC3243622 21926968

[B24] Llobet A. Gallop J. L. Burden J. J. E. Camdere G. Chandra P. Vallis Y. (2011). Endophilin drives the fast mode of vesicle retrieval in a ribbon synapse. *J. Neurosci.* 31 8512–8519 10.1523/JNEUROSCI.6223-09.2011 PMC3926091 21653855

[B25] LoGiudice L. Matthews G. (2007). Endocytosis at ribbon synapses. *Traffic* 8 1123–1128 10.1111/j.1600-0854.2007.00591.x 17547701

[B26] LoGiudice L. Sterling P. Matthews G. (2009). Vesicle recycling at ribbon synapses in the finely branched axon terminals of mouse retinal bipolar neurons. *Neuroscience* 164 1546–1556 10.1016/j.neuroscience.2009.09.023 PMC2784209 19778591

[B27] López-del Hoyo N. Fazioli L. López-Begines S. Fernández-Sánchez L. Cuenca N. Llorens J. (2012). Overexpression of guanylate cyclase activating protein 2 in rod photoreceptors in vivo leads to morphological changes at the synaptic ribbon. *PLoS ONE *7:e42994. 10.1371/journal.pone.0042994 PMC3418235 22912773

[B28] Lovas B. (1971). Tubular networks in the terminal endings of the visual receptor cells in the human, the monkey, the cat and the dog. *Z. Zellforsch.* 121 341–357 10.1007/BF00337638

[B29] Matthews G. Fuchs P. (2010). The diverse roles of ribbon synapses in sensory neurotransmission. *Nat. Rev. Neurosci.* 11 812–822 10.1038/nrn2924 PMC3065184 21045860

[B30] McMahon H. T. Boucrot E. (2011). Molecular mechanism and physiological functions of clathrin-mediated endocytosis. *Nat. Rev. Mol. Cell Biol.* 12 517–533 10.1038/nrm3151 21779028

[B31] Mercer A. J. Thoreson W. B. (2011). The dynamic architecture of photoreceptor ribbon synapses: cytoskeletal, extracellular matrix, and intramembrane proteins. *Vis. Neurosci.* 28 453–471 10.1017/S0952523811000356 PMC3437624 22192503

[B32] Mühlhans J. Brandstätter J. H Gießl A. (2011). The centrosomal protein pericentrin identified at the basal body complex of the connecting cilium in mouse photoreceptors. *PLoS ONE *6:e26496. 10.1371/journal.pone.0026496 PMC3198765 22031837

[B33] Nichols B. J. Lippincott-Schwartz J. (2001). Endocytosis without clathrin coats. *Trends Cell Biol.* 11 406–412 10.1016/S0962-8924(01)02107-9 11567873

[B34] Raimondi A. Ferguson S. M. Lou X. Armbruster M. Paradise S. Giovedi S. (2011). Overlapping role of dynamin isoforms in synaptic vesicle endocytosis. *Neuron* 70 1100–1114 10.1016/j.neuron.2011.04.031 21689597 PMC3190241

[B35] Rao-Mirotznik R. Harkins A. B. Buchsbaum G. Sterling P. (1995). Mammalian rod terminal: architecture of a binary synapse. *Neuron* 14 561–569 10.1016/0896-6273(95)90312-7 7695902

[B36] Rea R. Li J. Dharia A. Levitan E. S. Sterling P. Kramer R. H. (2004). Streamlined synaptic vesicle cycle in cone photoreceptor terminals. *Neuron* 41 755–766 10.1016/S0896-6273(04)00088-1 15003175

[B37] Regus-Leidig H Brandstätter J. H. (2012). Structure and function of a complex sensory synapse. *Acta Physiol. (Oxf.)* 204 479–486 10.1111/j.1748-1716.2011.02355.x 21880116

[B38] Regus-Leidig H. Ott C. Löhner M. Atorf J. Fuchs M. Sedmak T. (2013). Identification and immunocytochemical characterization of piccolino, a novel piccolo splice variant selectively expressed at sensory ribbon synapses of the eye and ear. *PLoS ONE* 8:e70373. 10.1371/journal.pone.0070373 PMC3735604 23936420

[B39] Regus-Leidig H. Specht D. tom Dieck S Brandstätter J. H. (2010a). Stability of active zone components at the photoreceptor ribbon complex. *Mol. Vis.* 16 2690–2700 PMC3002953 21179232

[B40] Regus-Leidig H. tom Dieck S Brandstätter J. H. (2010b). Absence of functional active zone protein Bassoon affects assembly and transport of ribbon precursors during early steps of photoreceptor synaptogenesis. *Eur. J. Cell Biol.* 89 468–475 10.1016/j.ejcb.2009.12.006 20188438

[B41] Regus-Leidig H. tom Dieck S. Specht D. Meyer L Brandstätter J. H. (2009). Early steps in the assembly of photoreceptor ribbon synapses in the mouse retina: the involvement of precursor spheres. *J. Comp. Neurol.* 512 814–824 10.1002/cne.21915 19067356

[B42] Royle S. J. Lagnado L. (2010). Clathrin-mediated endocytosis at the synaptic terminal: bridging the gap between physiology and molecules. *Traffic* 11 1489–1497 10.1111/j.1600-0854.2010.01104.x PMC3371399 20633242

[B43] Saheki Y de Camilli P. (2012). Synaptic vesicle endocytosis. *Cold Spring Harb. Perspect. Biol.* 4 a005645 10.1101/cshperspect.a005645 PMC3428771 22763746

[B44] Samorajski T. (1966). Structural organization of the retina in the tree shrew (Tupaia glis). *J. Cell Biol.* 28 489–504 10.1083/jcb.28.3.489 PMC2106936 5960809

[B45] Schmitz F. (2009). The making of synaptic ribbons: how they are built and what they do. *Neuroscientist* 15 611–624 10.1177/1073858409340253 19700740

[B46] Schneider C. A. Rasband W. S. Eliceiri K. W. (2012). NIH Image to ImageJ: 25 years of image analysis. *Nat. Methods* 9 671–675 10.1038/nmeth.2089 PMC5554542 22930834

[B47] Schwarz K. Natarajan S. Kassas N. Vitale N. Schmitz F. (2011). The synaptic ribbon is a site of phosphatidic acid generation in ribbon synapses. *J. Neurosci.* 31 15996–16011 10.1523/JNEUROSCI.2965-11.2011 PMC6623010 22049442

[B48] Sherry D. M. Heidelberger R. (2005). Distribution of proteins associated with synaptic vesicle endocytosis in the mouse and goldfish retina. *J. Comp. Neurol.* 484 440–457 10.1002/cne.20504 15770653

[B49] Spiwoks-Becker I. Vollrath L. Seeliger M. W. Jaissle G. Eshkind L. G. Leube R. E. (2001). Synaptic vesicle alterations in rod photoreceptors of synaptophysin-deficient mice. *Neuroscience* 107 127–142 10.1016/S0306-4522(01)00345-1 11744253

[B50] Sterling P. Matthews G. (2005). Structure and function of ribbon synapses. *Trends Neurosci.* 28 20–29 10.1016/j.tins.2004.11.009 15626493

[B51] Südhof T. C. Rizo J. (2011). Synaptic vesicle exocytosis. *Cold Spring Harb. Perspect. Biol.* 3 a005637 10.1101/cshperspect.a005637 PMC3225952 22026965

[B52] tom Dieck S. Altrock W. D. Kessels M. M. Qualmann B. Regus H. Brauner D. (2005). Molecular dissection of the photoreceptor ribbon synapse: physical interaction of Bassoon and RIBEYE is essential for the assembly of the ribbon complex. *J. Cell Biol.* 168 825–836 10.1083/jcb.200408157 PMC2171818 15728193

[B53] tom Dieck S Brandstätter J. H. (2006). Ribbon synapses of the retina. *Cell Tissue Res.* 326 339–346 10.1007/s00441-006-0234-0 16775698

[B54] Van Hook M. J. Thoreson W. B. (2012). Rapid synaptic vesicle endocytosis in cone photoreceptors of salamander retina. *J. Neurosci.* 32 18112–18123 10.1523/JNEUROSCI.1764-12.2012 PMC3547361 23238726

[B55] Wahl S. Katiyar R. Schmitz F. (2013). A local, periactive zone endocytic machinery at photoreceptor synapses in close vicinity to synaptic ribbons. *J. Neurosci.* 33 10278–10300 10.1523/JNEUROSCI.5048-12.2013 PMC6618599 23785143

[B56] Xi Q. Pauer G. J. T. Ball S. L. Rayborn M. Hollyfield J. G. Peachey N. S. (2007). Interaction between the photoreceptor-specific tubby-like protein 1 and the neuronal-specific GTPase dynamin-1. *Invest. Ophthalmol. Vis. Sci.* 48 2837–2844 10.1167/iovs.06-0059 PMC3021943 17525220

[B57] Yamada E. (1965). “Some observations on the membrane-limited structure within the retinal element,” in *Intracellular Membraneous Structure* eds Seno S. Cowdry E. V. (Okayama: Japan Society Cell Biology) 49–63

[B58] Zampighi G. A. Schietroma C. Zampighi L. M. Woodruff M. Wright E. M. Brecha N. C. (2011). Conical tomography of a ribbon synapse: structural evidence for vesicle fusion. *PLoS ONE* 6:e16944 10.1371/journal.pone.0016944 PMC3046965 21390245

